# Bevacizumab in combination with biweekly capecitabine and irinotecan, as first-line treatment for patients with metastatic colorectal cancer

**DOI:** 10.1038/sj.bjc.6605907

**Published:** 2010-10-26

**Authors:** P García-Alfonso, A J Muñoz-Martin, S Alvarez-Suarez, Y Jerez-Gilarranz, M Riesco-Martinez, P Khosravi, M Martin

**Affiliations:** 1 Medical Oncology service, Hospital General Universitario Gregorio Marañón, C/ Doctor Equerdo, 46, Madrid 28007, Spain

**Keywords:** bevacizumab, capecitabine, irinotecan, metastatic colorectal cancer, combined modality treatment

## Abstract

**Background:**

Combination of capecitabine and irinotecan (XELIRI regimen) is an active and well tolerated treatment for metastatic colorectal cancer (mCRC). The aim of this study was to evaluate the efficacy and safety of this regimen in combination with bevacizumab (BV), as first-line treatment for mCRC.

**Patients and methods:**

A total of 46 consecutive patients received a combination of BV (5 mg kg^−1^, day 1), irinotecan (175 mg m^−2^, day 1) and capecitabine (1000 mg m^−2^ twice daily on day 2–8), every 2 weeks. Patients were treated until disease progression or unacceptable toxicity. The primary objective was to determine the progression-free survival (PFS) and safety profile.

**Results:**

The overall response rate (ORR) was 67.4%, with a disease control rate (ORR+stable disease) of 93.5%. Median PFS and overall survival (OS) were 12.3 months (95% confidence interval (CI): 6.5–18.1 months) and 23.7 months (95% CI: 16.7–30.6 months), respectively. The most frequent grade 3/4 treatment-related adverse events were asthenia (7%), diarrhoea (7%), nausea (9%) and vomiting (7%).

**Conclusion:**

Bevacizumab combined with biweekly XELIRI is a highly active first-line regimen for mCRC treatment, showing encouraging PFS, ORR and OS with a good tolerability.

Colorectal cancer (CRC) is the third most commonly diagnosed malignancy and the second leading cause of cancer death in the world, resulting in >800 000 deaths every year. In 2006, CRC accounted for around 412 900 cases, 12.9% of all reported cases of cancer in Europe, being the second cause of cancer related death, with 207 400 (12.2%) deaths (Van Cutsem and Oliveira, 2009). Overall, up to 20% of patients have metastatic disease at the time of diagnosis, and ∼50–60% of patients will eventually develop metastatic or advanced disease, with a poor 5-year survival rate of 5%.

With the introduction in the last years of new regimens with biological agents and different combinations of chemotherapy, the progression-free survival (PFS) and overall survival (OS) have substantially improved. Although a variety of combination therapies have been studied and evaluated, the selection of the first-line treatment for metastatic colorectal cancer (mCRC) remains difficult, however, an optimum first-line approach currently to be used would be either 5-fluorouracil/leucovorin plus oxaliplatin or 5-fluorouracil/leucovorin plus irinotecan (FOLFIRI) combined with a biological agent such as bevacizumab (BV).

Bevacizumab, a recombinant human monoclonal antibody targeting vascular endothelial growth factor, was approved for first-line treatment of patients with advanced CRC by the EMEA, in early 2005, based on the data from the phase III AVF2107g trial (Hurwitz *et al*, 2004), which showed an increased response rate (RR), with an improved median duration of survival and a longer median PFS.

As its introduction, fluoropyrimidine-based chemotherapy has been the mainstay of chemotherapy treatment for CRC. Capecitabine is an oral fluoropyrimidine that has similar efficacy to 5-fluorouracil/leucovorin (5-FU/LV) as first-line treatment of advanced or mCRC (Hoff, *et al*, 2001; Van Cutsem *et al*, 2004), with the advantage of convenient oral administration and favourable safety profile (Scheithauer *et al*, 2003).

In xenograft models, the combination of BV and capecitabin resulted in a synergistic effect, with a greater duration of tumour inhibition than with either agent alone (Shen *et al*, 2004). Likewise, in several phase I and II trials it has been observed that capecitabine and irinotecan (XELIRI) can be equally effective, and safely combined in the most convenient alternative XELIRI regimen in patients with advanced CRC, with no pharmacokinetic interactions reported (Tewes *et al*, 2003; Bajetta *et al*, 2004).

In a previous clinical study on patients with mCRC, carried out by our group in a biweekly combination of irinotecan and capecitabine, was demonstrated that this schedule had a synergistic effect, with an acceptable RR of 32% and good tolerability as first-line treatment for mCRC, together with an important time to progression of 9 months and an OS of 19.2 months in this advanced setting. (Garcia-Alfonso *et al*, 2009).

Based on these premises, it is to be expected that the combination of BV with this biweekly XELIRI scheme would be at least as effective as the standard FOLFIRI regimen with an expected good safety profile. The objectives of this study were to evaluate the safety and the efficacy of this combination as first-line treatment in patients with mCRC.

## Patients and methods

Patients with histological or cytological confirmation of advanced CRC were treated with BV plus XELIRI as first-line treatment in one centre (Hospital General Universitario Gregorio Marañón, Madrid, Spain). Signed informed consent was obtained from all patients before starting treatment.

The primary objective was to determine the PFS, safety and tolerability to the biweekly BV–XELIRI regimen. Secondary objectives included overall response rate (ORR) and OS.

The study was conducted according to the Good Clinical Practices and Declaration of Helsinki, and the Institutional Review Board of the hospital approved this study.

### Patient eligibility

Patients diagnosed with initially unresectable chemotherapy-naïve mCRC were eligible. Main enrolment criteria were: age 18–75 years; presence of at least one unidimensionally measurable lesion; Eastern Cooperative Oncology Group (ECOG) performance status ⩽2; adequate bone marrow function (neutrophil count ⩾1.5 × 10^9^, platelets ⩾100 × 10^9^, haemoglobin ⩾10 g dl); serum creatinine <1.25 mg dl; alanine aminotransferase or aspartate aminotransferase or alkaline phosphatase <2.5 times the upper limit of normal or less than five times in the presence of liver metastases. Previous adjuvant chemotherapy must have been finished at least 6 months before enrolment in the study. Patients were excluded if they had brain metastases, had inadequately controlled hypertension, or had lack of physical integrity of the upper gastrointestinal tract or malabsorption syndrome. Because of the toxicities associated with BV, patients with myocardial infarction within 1 year before start of treatment, stroke, thromboembolic events, severe bleeding within 6 months before treatment, haemorrhagic diathesis, non-healing wounds or fractures or proteinuria ⩾500 mg/24 h in urine sample were excluded.

### Treatment protocol

Patients were treated with BV 5 mg kg^−1^ on day 1, followed by irinotecan 175 mg m^−2^ as a 30-min intravenous (i.v.) infusion on day 1 and capecitabine 1000 mg m^−2^ orally twice daily; from day 2 to 8. All patients received 5-HT_3_ inhibitors for emesis prophylaxis. Treatment was administered every 2 weeks and continued until disease progression, patient refusal, unacceptable toxicity or death.

Appropriate dose interruptions/reductions were implemented in the event of specific toxicities, depending on their nature and intensity. The next course of treatment only began when the neutrophil count was >1.5 × 10^9^, the platelet count was >100 × 10^9^, and any other treatment-related toxicities were less than or equal to grade 1; otherwise, treatment was withheld for up to 2 weeks. If adverse events did not improve to grade 0 or 1 after 3 weeks, patients were discontinued from study. For grade 2–3 hand–foot syndrome, the capecitabine treatment was withheld until a resolution to less than or equal to grade 1, the second time this toxicity occurs treatment was restarted with a 25% dose reduction.

### Study assessments

A screening assessment including medical history, physical examination and a chest X-ray was conducted within 2 weeks before starting the treatment. Within 7 days before starting the treatment, further assessments included vital signs, ECOG performance status and laboratory tests (haematology; blood chemistry including liver and renal function test; and urinalysis). A computerised tomography scan of chest, abdomen and pelvis was completed within 4 weeks of starting the treatment. The assessment of response was based on investigator reported measurements according to Response Evaluation Criteria in Solid Tumours guidelines (Therasse *et al*, 2000). CR or PR patients were required to undergo a confirmatory disease assessment at least 4 weeks later.

### Statistical considerations

Descriptive statistics were reported as proportion and medians. Progression-free survival was defined as the period from the date of the first dose of study treatment to the first observation of disease progression or death by any cause. The OS was calculated as the period from the date of the first cycle of treatment until death of any cause or until the date of the last follow-up, at which data point was censored. Survival analysis (PFS and OS) was estimated by the Kaplan–Meier method (Kaplan and Meier, 1958). Safety was assessed in terms of toxicity and evaluated according to the NCI-Common Toxicity Criteria for Adverse Events (NCI-CTCAE), version 3.0. All analyses were performed using SPSS 11.5 for Windows (Chicago, IL, USA).

## Results

A total of 46 consecutive patients treated with BV plus biweekly XELIRI were included. Baseline characteristics for the evaluable patients are summarised in Table 1. Median age was 64 years (range 39–80). In all, 45 (98%) patients had an ECOG performance status of <2 at baseline, half of patients had multiple sites of metastases and these were mostly located in the liver (65%).

### Toxicity and dose administration

A total of 586 cycles of chemotherapy plus BV were administered with a median of 12 cycles per patient (range 1–35). Overall, 41% (*n*=19) of patients required at least one dose reduction of capecitabine and in 50% (*n*=23) treatment delay was needed; dose of irinotecan was reduced in 37% (*n*=17) of patients and delayed in 52% (*n*=24). There were no dose reductions of BV, but in 54% of the patients the administration was delayed at least once. Treatment interruption because of toxicity caused by capecitabine, irinotecan and BV was required in two, four and four patients, respectively.

Treatment was well tolerated and most of the adverse events reported were mild (NCI-CTCAE grade 1 or 2). The main haematology and non-haematology toxicities are summarised in Table 2. The most common grade 3–4 toxicities were: nausea (8.7%), diarrhoea (6.5%), vomiting (6.5%), arterial thrombosis (4.3%), hand-foot skin reaction (2.2%), hypertension (2.2%), intestinal obstruction (2.2%), neutropenia (2.2%) and hyperbilirubinemia (2.2%). Two cases of pulmonary thromboembolism led to continuation of therapy only without BV. No treatment-related deaths were reported.

### Efficacy and survival

**Figure 1:**
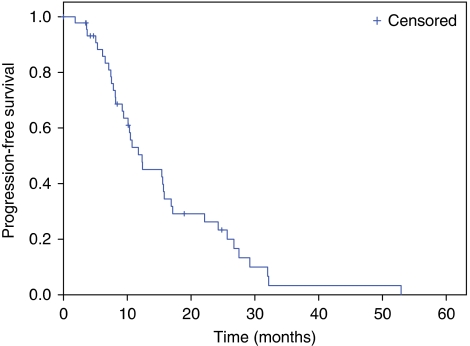
Kaplan–Meier estimates of progression-free survival.

**Figure 2:**
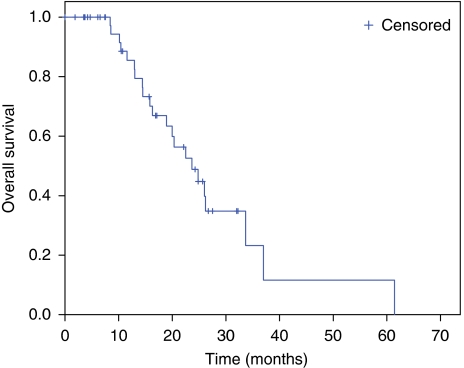
Kaplan–Meier estimates of overall survival.

The BV plus biweekly XELIRI regimen led to an objective response in 31 out of 46 (67%) patients of whom 4% had a CR and 63% had a PR. A further 12 (26%) patients had stable disease resulting in a disease control rate of 93% (Table 3). In this initially unresectable population, 13 patients (28%) underwent surgical removal of metastases after the treatment with chemotherapy plus BV, with curative intention in 4 patients with liver involvement only and in 2 with extrahepatic metastases; 7 patients were able to undergo a secondary tumour resection in terms of palliative therapy. The median time elapsed between the last administration of BV and surgery was 10.4 weeks. After a median follow-up of 15.8 months, 37 (80%) patients had progressed and the median duration of PFS was 12.3 months (95% confidence interval (CI): 6.5–18.1; Figure 1). So far, 23 (50%) patients have died, and median OS is 23.7 months (95% CI: 16.7–30.6 months; Figure 2).

## Discussion

Nowadays, there are multiple options for the treatment of mCRC that include different chemotherapeutic agents and biologics. Great effort has been made over the last years to improve the efficacy and safety of the chemotherapy combinations and to find new alternatives by combining the new biological agents with the classic regimens.

The addition of BV, a novel antiangiogenic drug, to the irinotecan/5-fluorouracil/leucovorin scheme (IFL regimen) in first-line treatment of mCRC has been reported to improve OS, PFS, objective RR and duration of response compared with IFL alone (Hurwitz *et al*, 2004). Recently, Fuchs *et al* (2008) reported that infusional 5-FU/LV with irinotecan and BV conferred a statistically significant OS when compared with bolus irinotecan and 5-FU/LV (modified IFL; 28.0 months *vs* 19.2 months; *P*=0.037; HR for death=1.79; 95% CI: 1.12–2.88), with diarrhoea as the main common dose-limiting toxicity.

Data from several trials have shown that 5-FU/LV regimen could be substituted by the orally administered fluoropyrimidine capecitabine when capecitabine-based combinations have been compared with the equivalent 5-FU/LV-based regimens. In terms of OS, capecitabine is at least equivalent to 5-FU/LV with the added benefits of convenience and a favourable safety profile. A phase III trial with previously untreated mCRC patients randomised to either oral capecitabine or i.v. bolus 5-FU/LV (Mayo Clinic regimen) demonstrated a significantly superior RR of capecitabine compared with 5-FU/LV (26 *vs* 17% *P*<0.0002). Overall survival in the two treatment arms was equivalent (median of 12.9 months with capecitabine *vs* 12.8 months with 5-FU/LV; Van Cutsem *et al*, 2004). The combination of capecitabine with irinotecan (XELIRI) has been shown to be more active than either drug alone. In our previous phase II study, in which patients with mCRC received capecitabine at 1000 mg m^−2^ twice daily (days 2–8) plus irinotecan at 175 mg m^−2^ (day 1) in a 15-day cycle, XELIRI combination yielded a 32% ORR and a median time to tumour progression of 9.9 months. Disease control was achieved in 66% of evaluable patients. The XELIRI regimen was relatively well tolerated, with the most common grade 3/4 toxicities being diarrhoea (15%) and neutropenia (8%) (Garcia-Alfonso *et al*, 2009). The fact that capecitabine is generally well tolerated makes it suitable for combination with other cytotoxics. Cassidy *et al* (2004), found that capecitabine plus oxaliplatin achieved consistently high (>50%) RRs across all patient sub-populations.

In spite of these encouraging results, in the BICC-C (Bolus, Infusional, or Capecitabine with Camptosar-Celecoxib) and EORTC 40015 phase III trials of XELIRI regimens, with and without targeted-agents, some concern about toxicity arose because of the greater proportion of GI events on the XELIRI arm (Fuchs *et al*, 2007; Köhne *et al*, 2008).

The standard schedule for capecitabine as monotherapy is 1250 mg m^−2^ twice daily for 14 days followed by 7 days off (14/7), however, from mathematical models and breast cancer xenografts studies, it is suggested that the greatest effect is achieved after ∼7 days of treatment and that a continued administration longer than 7 days adds toxicity, but no additional efficacy. Kolinsky *et al*, carried out an experimental study to determine the antitumor activity and tolerability of capecitabine administered using either a schedule of 7 days of treatment followed by 7 days drug-free (7/7) or 14/7 days, alone and in combination with irinotecan and BV. The authors concluded that the addition of BV to XELIRI significantly improved tumour growth inhibition and life span in the model studied and that modified capecitabine schedule improved the efficacy of doublet and triplet combinations without adding toxicity (Traina *et al*, 2008; Kolinsky *et al*, 2009).

To date, there have been limited data about the XELIRI plus BV regimen. Chen *et al* (2009), presented the preliminary results from a study using BV with irinotecan plus capecitabine, and they showed that this combination had promising clinical activity. They found an ORR of 40%, with an overall disease control rate of 86% and a 1-year progression-free rate of 49%.

At the 2009 Annual Meeting of the American Society of Clinical Oncology (ASCO), Ducreux *et al* (2009), reported the preliminary results of the phase II, non-comparative, randomised FNCLCC ACCORD 13/0503 trial, in which a total of 145 patients, from 18 to 72 years of age, were randomised to receive either BV plus XELIRI (irinotecan 200 mg m^−2^ on day 1, capecitabine 1000 mg m^−2^ twice daily on days 1–14 plus BV 7.5 mg kg^−1^ on day 1, every 3 weeks) or BV plus FOLFIRI (irinotecan 180 mg m^−2^ on day 1 plus 5-FU 400 mg m^−2^ plus leucovorin 400 mg m^−2^ on day 1 followed by 5-FU 2400 mg m^−2^ as a 46-hour infusion plus BV 5 mg kg^−1^ on day 1, every 2 weeks). The preliminary results from the first 6-month follow-up reported an ORR of 58% (95% CI: 47–70%) in the BV plus XELIRI arm similar to 58% (95% CI: 53–65%) in the BV plus FOLFIRI arm. The most common grade 3–4 adverse events reported in the XELIRI and FOLFIRI groups were neutropenia (17 *vs* 26%), diarrhoea (12 *vs* 5%) and cardiovascular events (13 *vs* 11%). The investigators concluded that XELIRI and FOLFIRI plus BV are both similarly effective in the treatment of patients with mCRC, with manageable toxicity.

In this single-institutional study, with the combination of biweekly XELIRI plus BV for previously untreated mCRC patients, we observed a meaningful clinical activity, with an ORR of 67.4%, a median PFS of 12.3 months, and a median OS of 23.7 months. The overall disease control rate was 93.5%. In general, this combination was relatively well tolerated, with most of the adverse events being grade 1–2. Interestingly, the overall safety profile of this combination differs from those reported with the XELIRI regimen by Fuchs. In the BICC-C trial, the XELIRI arm was associated with a significantly higher incidence of grade 3/4 diarrhoea (48%), neutropenia (32%) and dehydration (19% Fuchs *et al*, 2007). In the 40015 clinical trial conducted by EORTC group, XELIRI was associated with increased mortality as well as a nearly 40% incidence of grade 3/4 diarrhoea (Köhne *et al*, 2008). In these two clinical trials, the increased toxicity clearly impacted the clinical activity of the XELIRI regimen in a negative manner. However, it is noteworthy that the doses of XELIRI used in these studies were higher than the ones used in this XELIRI plus BV combination.

Compared with the recent findings of the Ducreux's trial, which investigated the combination of XELIRI and FOLFIRI along with BV, the clinical activity of the data here reported in terms of ORR was similar to what has been reported for the FNCLCC ACCORD 13/0503 trial, with a better toxicity profile, possibly due to the lower dose of chemotherapeutic agents in this XELIRI plus BV regimen.

Our analyses show that the combination of BV with the XELIRI regimen is feasible with manageable toxicity, and that is associated with a promising efficacy in terms of PFS, ORR and OS in previously untreated mCRC.

## Conclusion

In summary, this study suggests that a biweekly schedule of capecitabine plus BV is a promising regimen that should be considered as a one of the front-line standards of care for patients with mCRC.

## Figures and Tables

**Table 1:** Baseline patient characteristics

**Characteristic**	**Number of patients (%)**
Patients	46
Median age (years; range)	64 (39–80)
	
*Sex*	
Male	21 (46)
Female	25 (54)
	
*ECOG PS*	
0	7 (15)
1	38 (83)
2	1 (2)
	
*Previous therapy*	
Surgery	16 (35)
Radiation	14 (30)
Chemotherapy	12 (26)
	
*Number of metastatic sites*	
1	20 (43.5)
2	20 (43.5)
⩾3	6 (13)
	
*Metastatic site(s)*	
Liver	30 (65)
Lung	18 (39)
Lymph node(s)	17 (37)
Peritoneum	6 (13)
Other	7 (15)

Abbreviation: ECOG PS=Eastern Cooperative Oncology Group performance status.

**Table 2:** Most common treatment-related adverse events per patient

	**Grade 1/2**		**Grade 3/4**		**Total**	
	***N***	**%**	***N***	**%**	***N***	**%**
Diarrhoea	29	63	3	7	32	70
Asthenia	28	61	3	7	31	67
Nausea	26	57	4	9	30	65
Vomiting	22	48	3	7	25	54
Alopecia	19	41	6	13	25	54
Mucositis	20	44	1	2	21	46
Anaemia	20	43.5	—	—	20	44
Hand–foot skin reaction	17	37	1	2	18	39
Bleeding	17	37	—	—	17	37
Constipation	7	15	2	4	9	20
Neutropenia	6	13	1	2	7	15
Proteinuria	6	13	—	—	6	13
Epigastralgia	6	13	—	—	6	13
Anorexia	5	11	—	—	5	11
Headache	5	11	—	—	5	11
Fever	5	11	—	—	5	11

**Table 3:** Best response to treatment

	**XELIRI plus bevacizumab (%)**
Complete response	2 (4)
Partial response	29 (63)
Objective response	31 (67)
Progressive disease	12 (6.5)
R0 resection	6 (13)

Abbreviation: XELIRI=capecitabine and irinotecan.
